# Chloroquine Potentiates Primaquine Activity against Active and Latent Hepatic Plasmodia *Ex Vivo*: Potentials and Pitfalls

**DOI:** 10.1128/AAC.01416-20

**Published:** 2020-12-16

**Authors:** Laurent Dembélé, Jean-François Franetich, Valérie Soulard, Nadia Amanzougaghene, Shahin Tajeri, Teun Bousema, Geert-Jan van Gemert, Roger Le Grand, Nathalie Dereuddre-Bosquet, J. Kevin Baird, Dominique Mazier, Georges Snounou

**Affiliations:** a Sorbonne Université, INSERM, CNRS, INSERM U1135, Centre d’Immunologie et des Maladies Infectieuses, CIMI-Paris, Paris, France; b Université des Sciences, des Techniques et des Technologies de Bamako (USTTB), Bamako, Mali; c Malaria Research and Training Centre (MRTC), Faculty of Pharmacy, Bamako, Mali; d Department of Medical Microbiology, Radboud University Nijmegen Medical Center, Nijmegen, The Netherlands; e Université Paris-Saclay, Inserm, CEA, Center for Immunology of Viral, Auto-Immune, Hematalogical and Bacterial Diseases (IMVA-HB/IDMIT), Fontenay-aux-Roses, France; f Eijkman-Oxford Clinical Research Unit, Jakarta, Indonesia; g Centre for Tropical Medicine and Global Health, Nuffield Department of Medicine, University of Oxford, Oxford, United Kingdom

**Keywords:** chemotherapy, chloroquine, hepatic parasites, latent infection, malaria, potentiation, primaquine

## Abstract

For a long while, 8-aminoquinoline compounds have been the only therapeutic agents against latent hepatic malaria parasites. These have poor activity against the blood-stage plasmodia causing acute malaria and must be used in conjunction with partner blood schizontocidal agents. We examined the impacts of one such agent, chloroquine, upon the activity of primaquine, an 8-aminoquinoline, against hepatic stages of Plasmodium cynomolgi, Plasmodium yoelii, Plasmodium berghei, and Plasmodium falciparum within several *ex vivo* systems—primary hepatocytes of Macaca fascicularis, primary human hepatocytes, and stably transformed human hepatocarcinoma cell line HepG2. Primaquine exposures to formed hepatic schizonts and hypnozoites of P. cynomolgi in primary simian hepatocytes exhibited similar 50% inhibitory concentration (IC_50_) values near 0.4 μM, whereas chloroquine in the same system exhibited no inhibitory activities. Combining chloroquine and primaquine in this system decreased the observed primaquine IC_50_ for all parasite forms in a chloroquine dose-dependent manner by an average of 18-fold. Chloroquine also decreased the primaquine IC_50_ against hepatic P. falciparum in primary human hepatocytes, P. berghei in simian primary hepatocytes, and P. yoelii in primary human hepatocytes. Chloroquine had no impact on primaquine IC_50_ against P. yoelii in HepG2 cells and, likewise, had no impact on the IC_50_ of atovaquone (hepatic schizontocide) against P. falciparum in human hepatocytes. We describe important sources of variability in the potentiation of primaquine activity by chloroquine in these systems. Chloroquine potentiated primaquine activity against hepatic forms of several plasmodia. We conclude that chloroquine specifically potentiated 8-aminoquinoline activities against active and dormant hepatic-stage plasmodia in normal primary hepatocytes but not in a hepatocarcinoma cell line.

## Abstract (precis)

For a long while, 8-aminoquinoline compounds have been the only therapeutic agents against latent hepatic malaria parasites. These have poor activity against the blood-stage plasmodia causing acute malaria and must be used in conjunction with partner blood schizontocidal agents. We examined the impacts of one such agent, chloroquine, upon the activity of primaquine, an 8-aminoquinoline, against hepatic stages of Plasmodium cynomolgi, Plasmodium yoelii, Plasmodium berghei, and Plasmodium falciparum within several *ex vivo* systems—primary hepatocytes of Macaca fascicularis, primary human hepatocytes, and stably transformed human hepatocarcinoma cell line HepG2.

## INTRODUCTION

The dormant hepatic forms of the human malaria parasites Plasmodium vivax and Plasmodium ovale, called hypnozoites, awaken from latency within weeks to several years to cause often multiple clinical attacks of acute malaria known as relapses. Each incapacitating attack carries the risk of severe morbidity and onward transmission. Among the dozens of antimalarial drugs, only three 8-aminoquinolines have proven efficacy against hypnozoites. Plasmochin, first introduced in the 1920s, was supplanted by primaquine in the early 1950s, and in turn with tafenoquine, which was only licensed in 2018 (1). The propensity of 8-aminoquinolines to provoke life-threatening hemolysis in glucose-6-phosphate dehydrogenase (G6PD)-deficient patients imposes the necessity of laboratory screening and clinical supervision. These services are usually unavailable where most malaria patients live. Less toxic hypnozoitocidal therapies suitable for routine administration would greatly improve the control and elimination of these insidiously harmful parasites.

How 8-aminoquinolines destroy hypnozoites remains unknown. Primaquine appears to be a prodrug, with one or several active metabolites generated by the cytochrome P450 2D6 (CYP2D6) enzyme (1), but direct evidence informing these molecular mechanics has yet to be demonstrated. As such, the search for better compounds of this class has always been and remains largely empirical. Assessment of efficacy against hypnozoites has largely relied either on human subjects (plasmochin and primaquine) or on *in vivo* infections of laboratory macaques (tafenoquine) by the relapsing species Plasmodium cynomolgi, a hypnozoite-bearing species closely related to P. vivax. Advances in the *ex vivo* cultivation of primary hepatocytes (human and simian) have opened the way for investigating hypnozoites and their biology (2, 3), medium-throughput screening for hypnozoitocidal compounds, and definitive exploration of the elusive mechanism of 8-aminoquinoline activity against latent malaria (4–7).

Hypnozoitocidal 8-aminoquinoline therapies must be combined with effective blood schizontocidal antimalarials for what is often called radical cure, i.e., terminating both latency and the acute attack, respectively. Chloroquine and primaquine radical cure of P. vivax has been standard therapy since 1952. Although chloroquine (or quinine) alone has no discernible activity against hypnozoites, an extraordinary phenomenon has been consistently observed in early clinical trials combining these drugs with 8-aminoquinolines against P. vivax and P. cynomolgi; each powerfully potentiated the hypnozoitocidal activity of 8-aminoquinolines (8, 9). This phenomenon has also been observed for tafenoquine in Rhesus monkeys challenged with P. cynomolgi (10). An understanding of this hitherto unexplored phenomenon carries the potential to optimize the potentiation of 8-aminoquinolines so as to minimize the dose and thus the risk to G6PD-deficient patients (11), while perhaps also illuminating otherwise elusive mechanisms of action. However, such investigations *in vivo* have been prohibitively costly, laborious, or ethically challenging. We have examined the potential for an *ex vivo* system to serve as a practical means of conducting detailed cellular and molecular investigations of the phenomenon of potentiated 8-aminoquinoline therapeutics by chloroquine.

## RESULTS AND DISCUSSION

**FIG 1:**
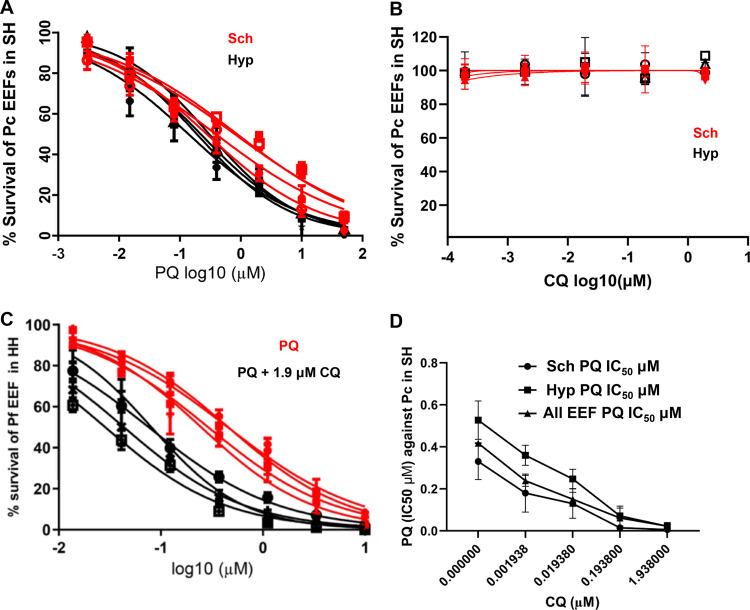
Chloroquine potentiation of primaquine activity on P. cynomolgi hepatic-stage parasites. (A and B) Survival of P. cynomolgi exo-erythrocytic forms (EEFs), hepatic schizonts (Sch), or putative hypnozoites (Hyp) in primary simian hepatocytes following exposure (D5-D8) to (A) primaquine (PQ) or (B) chloroquine (CQ). Control wells (no drug) harbored 101 EEF, of which ca. 58 were Sch and 43 were Hyp on the day the cultures were fixed (D8) (error bars represent ± standard error). (C) Dose response curve of PQ (*x* axis) activity on the hepatic forms of P. cynomolgi (EEF) in primary simian hepatocytes (SH) in the presence or absence of a fixed CQ concentration. An average of 101 EEFs were observed in each of the control wells (error bars represent ± standard deviation). (D) Dose response curve of PQ IC_50_ on total EEFs, schizonts, or putative hypnozoites of P. cynomolgi in primary simian hepatocytes (error bars represent ± standard error). The data presented for P. cynomolgi in panels A to D are derived from four independent biological replicates in which each point was derived from data from 3 replica wells. The batches of primary simian hepatocytes were derived from two monkeys, with two of the biological replicates conducted with one batch and the other two with the other batch.

**FIG 2:**
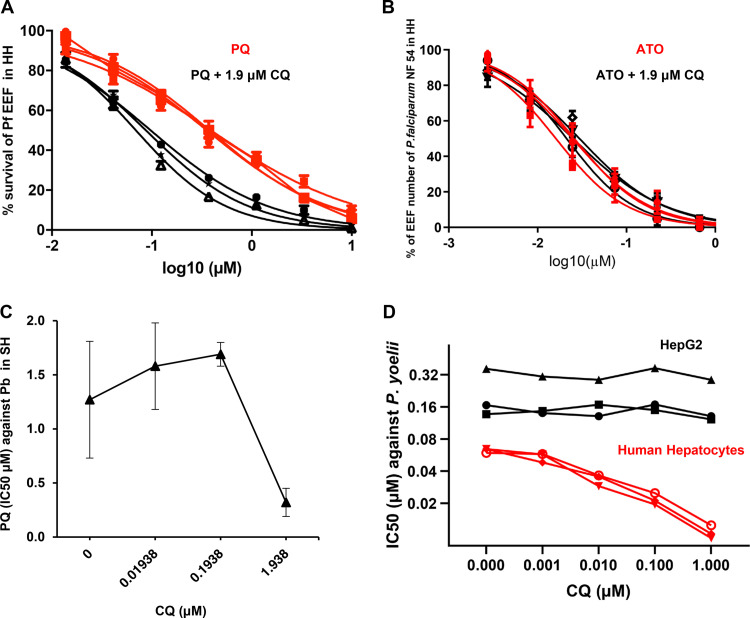
Chloroquine potentiation of primaquine activity on the hepatic stages of other parasite species. (A) Dose response curve of PQ (*x* axis) activity on the hepatic forms of P. falciparum (EEF) in primary human hepatocytes in the presence or absence of a fixed CQ concentration (exposed from D4 to D7 post-sporozoite inoculation). An average of 144 EEFs were observed in each of the control wells. (B) Survival of P. falciparum NF54 exo-erythrocytic forms (EEFs) in primary human hepatocytes following exposure to a serial dilution of atovaquone (ATO) alone or ATO combined with a fixed dose of CQ (exposed from D4 to D7 post-sporozoite inoculation). The data presented for P. falciparum are derived from three independent biological replicates in which each point was derived from three replica wells (error bars represent ± standard deviation). (C) Dose response curve of PQ IC50 on the hepatic forms (EEF) of P. berghei in primary simian hepatocytes (from batch SH30093L2) in the presence of increasing concentrations of CQ. An average of 130 EEFs to 150 EEFs were observed in each of the control wells (exposed from 3 h to 48 h post-sporozoite inoculation). The data presented for P. berghei are derived from an experiment in which each point was derived from four replica wells (error bars represent ± standard error). (D) Variation in the IC50 of PQ on the hepatic forms (EEF) of P. yoelii in the presence of increasing concentrations of CQ, either in primary human hepatocytes (HH) or in the HepG2 hepatocarcinoma line (exposed from 3 h to 48 h post-sporozoite inoculation). An average of 187 EEFs in HH, and 228 EEFs in HepG2 were observed in each of the control wells. The data presented for P. yoelii are derived from three independent biological replicates in which each point was derived from three replica wells (error bars represent ± standard deviation) of two technical replicates (error bars represent ± standard deviation). The human primary hepatocytes used for the P. falciparum and P. yoelii experiments were derived from three distinct donors.

In the first experiment, the possible potentiation of primaquine (PQ) by chloroquine (CQ) was examined in *ex vivo* cultured primary Macaca fascicularis hepatocytes infected by P. cynomolgi. Eight days post-sporozoite inoculation, two types of exo-erythrocytic forms (EEFs) were observed in the cultures, large multinucleated schizonts (Schz) and relatively small uninucleated forms (Hyp) we considered to be hypnozoites (2, 3). Exposure to PQ alone (days 5 to 8 postinoculation) exerted dose-dependent inhibitory activity against both parasite forms (Fig. 1A), whereas similar exposure to CQ alone had no effect on parasite numbers or development (Fig. 1B). When measured in the presence of increasing CQ doses, the PQ 50% inhibitory concentration (IC_50_) values decreased significantly (*P* < 0.0001) in a dose-dependent manner against all the P. cynomolgi hepatic forms (Fig. 1C and D, supplemental figures). Potentiation of PQ activity by CQ was similarly observed for Plasmodium falciparum in *ex vivo* cultured human primary hepatocytes (Fig. 2A). In similar experiments, CQ had no impact on the activity of atovaquone, a drug that eliminates maturing hepatic stages but not hypnozoites (Fig. 2B). We further examined the potentiation phenomenon employing the rodent malarias, Plasmodium berghei and Plasmodium yoelii. In these experiments we opted to use primary hepatocytes from macaques and humans, respectively, in order to preclude any variations in PQ metabolism that might be inherent to rodent primary hepatocytes. The activity of PQ against hepatic stages of P. berghei infecting *ex vivo* cultured *M. fascicularis* primary hepatocytes was enhanced by CQ (Fig. 2C, supplemental figures). However, this observation was derived from a single experiment, which would merit repeating in order to determine whether the different dose-dependent potentiation pattern is due to the hepatocyte batch or inherent to the parasite species. We then exploited the ability of P. yoelii to infect and mature in both human primary hepatocytes and in a hepatocarcinoma cell line (HepG2-A16) (12) lacking cytochrome P450 activities. Significant potentiation of PQ by CQ occurred in P. yoelii-infected human primary hepatocytes, but not in HepG2-A16 cells (Fig. 2D, supplemental figure).

These observations demonstrated potent potentiation of PQ activity against hepatic *Plasmodium* parasites by CQ, whether observed in P. cynomolgi infecting *ex vivo* macaque hepatocyte cultures as active schizonts or dormant hypnozoites or in three other nonrelapsing *Plasmodium* species. In contrast, potentiation was not observed for a *Plasmodium*-infected hepatocarcinoma cell line (HepG2) lacking CYP2D6 and other cytochrome activities. Whereas P. berghei in *M. fascicularis* primary hepatocytes might appear to be the most convenient experimental *ex vivo* model, investigations aimed at assessing or optimizing activity against hypnozoites require infection by P. cynomolgi sporozoites, since potent prophylactic inhibitory activity against the hepatic stages is not necessarily predictive of radical cure activity that specifically targets hypnozoites (13).

**FIG 3:**
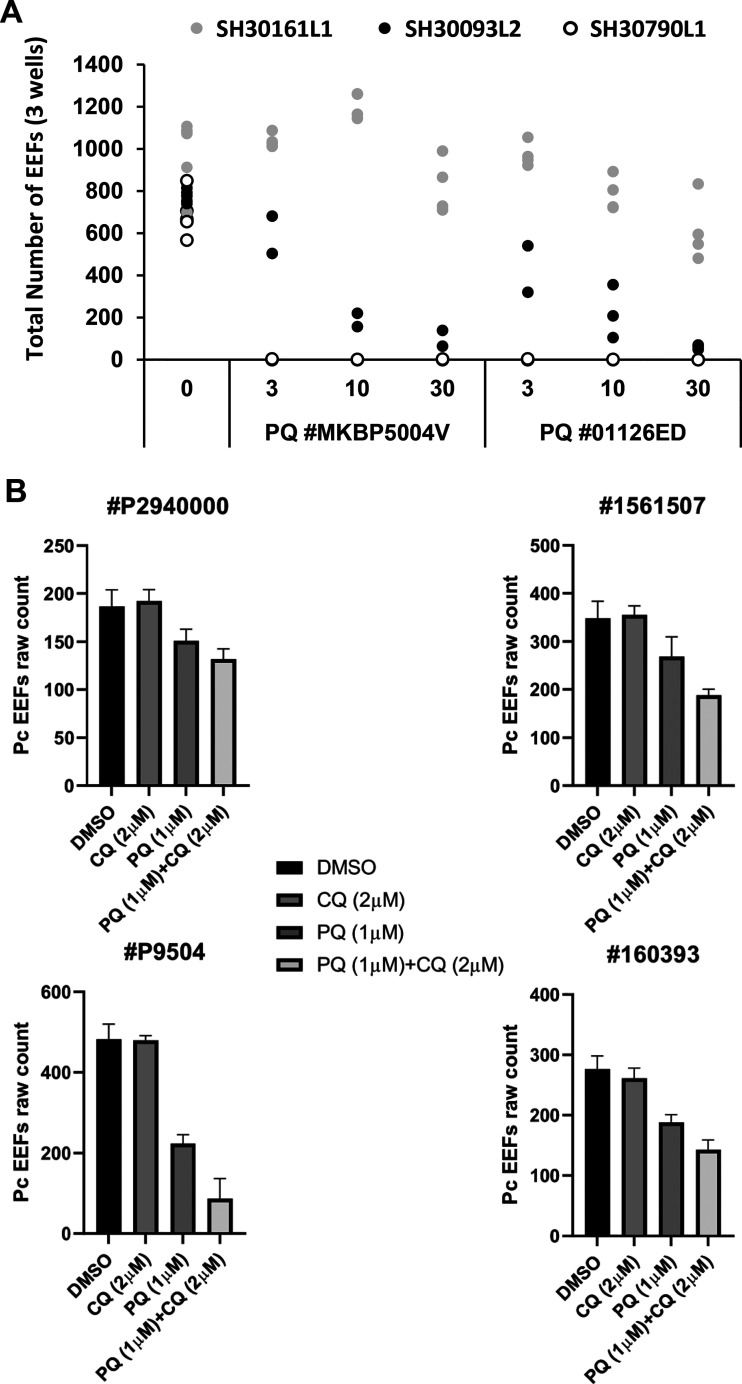
Variability of the potentiation phenomenon. (A) Number of PbGFP EEFs in infected cultures treated with two batches of primaquine (PQ number MKBP5004V and PQ number 01126ED) at 3, 10, or 30 μM, from 3 h postinfection to fixation at D2. The data are derived from an experiment in which each dot represented the total number of parasites counted per replica well for the three distinct batches of simian primary hepatocytes isolated from three different *M. fascicularis* monkeys as follows: SH30161L1, gray dots; SH30093L2, black dots; and SH30790L1, empty lank circles. In the set of experiments with SH30161L1, 10 μM methylene blue was also present; nonetheless, this compound has no activity against the hepatic stages (14). (B) Simian primary hepatocytes isolated from the liver of a single *M. fascicularis* were thawed at different times and then infected with four distinct batches of P. cynomolgi sporozoites. Infected cultures were treated from D5 to D8 post-sporozoite inoculation with 1 μM primaquine (batches numbers P9504, P2940000, 160393, and 1561507) with or without 2 μM chloroquine. DMSO was used in “no drug” control wells. The data presented for each PQ batch are derived from an independent experiment in which each point represents the mean number of P. cynomolgi hepatic parasites counted in the three replica wells (error bars represent ± standard deviation).

Nonetheless, we express caution regarding important pitfalls of these models. We observed recurring inconsistency in reproducing the potentiation phenomenon *ex vivo* due to an as yet unexplained variability in PQ activity. This was initially noted for the P. cynomolgi infections (see supplemental figures for the variations observed in the four experiments conducted with the same PQ lot). Furthermore, the susceptibility of P. berghei parasites to two distinct lots of PQ profoundly and similarly differed between *ex vivo* infections of thawed primary hepatocytes collected from three macaques (Fig. 3A, supplemental figure). In another independent set of experiments, robust potentiation of PQ activity by CQ was observed for only one of the four distinct PQ lots tested against *ex vivo* cultures of P. cynomolgi in thawed lots of primary hepatocytes originally obtained from the same macaque (Fig. 3B). Given the known unpredictable viability of both fresh and thawed cultured primary hepatocytes, taken with the naturally variable CYP2D6 activities impacting PQ activity, we suggest that the most likely explanation for the observed inconsistencies involves both the natural and induced metabolic and cytochrome activities of these *ex vivo* hepatocytes on, and subsequent to, drug exposure. Variations in response to PQ have been observed independently (5) and have been ascribed to differences in hepatocyte viability. More positively, such variability offers hitherto unavailable opportunities for demonstrating hepatocyte factor(s) acting as defining determinants of antiparasitic activity of PQ. That understanding, in turn, may directly inform strategies for elucidating the molecular basis of its potentiation by CQ.

Detailed analysis in human volunteers revealed complex pathways that generate numerous PQ metabolites (14), with the production of some but not others by *ex vivo* cultured hepatocytes influenced by the concurrent presence of CQ (15). The decrease of the CYP2D6-dependent PQ metabolites by CQ may favor the production of other active metabolites. It has been shown that coadministration of CQ increased the plasma concentration of PQ and some of its metabolites (16); thus, another possibility could be that chloroquine might also target other metabolic pathways to yield PQ metabolites active against the intrahepatic parasite, or that as a lysosomotropic agent, CQ might affect acid cell compartments. Further experimental work is required to fully dissect the molecular mechanism underlying the potentiation of PQ activity by CQ against hepatic malaria parasites, both active and dormant.

With such knowledge, potentiation of 8-aminoquinoline hypnozoitocidal activity by CQ or any other compounds to such an extent as to greatly mitigate or eliminate the hemolytic toxicity of these compounds may be possible. This would require the 8-aminoquinoline metabolite(s) involved in killing hypnozoites to differ from those responsible for hemolytic toxicity, either in chemical structure or by differential modifications of their relative concentrations to active but nontoxic levels. In any event, the *ex vivo* model system described here apparently captures the defining potentiation of PQ by CQ demonstrated previously only in patients or macaques. Variations of condition profoundly impacting that activity point the way to dissection of the molecular mechanics governing it and, ultimately, rationally optimizing the phenomenon to improve chemotherapeutics against latent malaria.

## MATERIALS AND METHODS

### Drugs.

Primaquine diphosphate salt and chloroquine diphosphate salt were purchased from Sigma-Aldrich (St. Louis, MO, USA) (reference numbers 160393-1g and C6628, respectively); atovaquone was purchased from Laboratoire GlaxoSmithKline (France).

### Parasites.

**P. cynomolgi (M strain).** Infected Macaca fascicularis or Macaca mulatta blood infectious to mosquitoes was obtained from the Division of Immuno-Virology, Institute of Emerging Diseases and Innovative Therapies, Commissariat à l’Energie Atomique et aux Energies Alternatives, Fontenay-aux-Roses, France. This was used to infect laboratory-bred Anopheles stephensi mosquitoes. Briefly, plasma and buffy coat were removed by centrifugation, and the pelleted red blood cells (RBC) were then resuspended in homologous serum or human AB plasma at 50% hematocrit. Throughout, the temperature was maintained at 37°C in order to preserve gametocyte infectivity. The mosquitoes were allowed to feed for 1 h using a Hemotek membrane feeding system and then maintained at 26°C at 70% humidity with 10% sucrose supplemented with 0.05% para-aminobenzoic acid until salivary gland dissection. The sporozoites were recovered from the infected salivary glands 14 to 30 days later as described elsewhere (2, 3).

**P. falciparum (strain NF54).** Live infected A. stephensi mosquitoes were obtained from the Department of Medical Microbiology, University Medical Centre St. Radboud, Nijmegen, Netherlands. The sporozoites were harvested as described above 14 to 21 days after the infective blood meal.

**P. berghei (strain PbGFP ANKA).** Sporozoites were from A. stephensi mosquitoes that had directly fed 14 to 21 days earlier on anesthetized mice infected with P. berghei PbGFP ANKA (17).

**P. yoelii*yoelii* (strain 265BY).** Sporozoites were obtained from A. stephensi mosquitoes that had directly fed 14 to 21 days earlier on anesthetized mice infected with P. yoelii *yoelii* 265BY.

### Hepatocytes.

Adult cynomolgus macaques (Macaca fascicularis) were imported from Mauritius and housed in the Infectious Disease Models for Innovative Therapies (IDMIT) facilities at the Fontenay-aux-Roses research center of the Commissariat d’Energie Atomique (CEA). They were used at the IDMIT in accordance with the French national regulation, under the supervision of national veterinary inspectors. IDMIT facilities are in compliance with the standards for human care and use of laboratory animals (Animal Welfare Assurance, OLAW number A5826-01). The use of nonhuman primates at CEA is in accordance with recommendations of the Weatherall report. Experimental procedures were conducted in strict accordance with the recommendations of the European guidelines for the care and use of laboratory animals (European directive 63/210). The protocols and the use of hepatocytes for the purpose of the work described here were approved by the Ethical Animal Committee of the CEA (permit number A 92-032-02). Simian primary hepatocytes were obtained from segments of liver collected from healthy long-tailed macaques (*M. fascicularis*) and then cryopreserved.

Human primary hepatocytes were either isolated from segments of liver taken from adult patients in the course of partial hepatectomy at the Service de Chirurgie Digestive, Hépato-Bilio-Pancréatique et Transplantation Hépatique, Hôpital Pitié-Salpêtrière, Paris, France, or purchased as cryopreserved cells from BioPredic International (BPI), France. The same procedure (18) was employed for the isolation of the simian and human primary hepatocytes. Viable human primary hepatocytes isolated from liver segments were immediately cultured and then used for sporozoite infections and drug testing, while those obtained commercially were thawed and then allowed to recover in culture for 4 days before infection and drug testing. Both human and simian hepatocytes were cultured as previously reported (3). HepG2-A16-CD81EGFP (HepG2) cells stably transformed to express a green fluorescent protein (GFP)-CD81 fusion protein were cultured as described previously (12).

### Hepatocyte infections.

P. cynomolgi, P. falciparum, P. berghei, or P. yoelii sporozoites were resuspended in the complete medium used for hepatocyte culture supplemented with 0.25 μg/ml amphotericin B (Gibco). The complete medium was as follows: William’s medium E (Life Technologies) supplemented with 10% fetal clone II serum (Fisher Scientific; SH30066.03), 5 × 10^−5^ M water-soluble hydrocortisone (Sigma; H0396), 5 μg per ml insulin (Sigma), 2 mM l-glutamine, and 200 U per ml penicillin, along with 200 μg per ml streptomycin (Life Technologies). Cultured simian and human hepatocytes were inoculated, respectively, with 30,000 P. cynomolgi or P. falciparum sporozoites per well (96-well plates) in a total volume of 50 μl. Thawed primary human hepatocytes were inoculated with 6,000 sporozoites per well (384-well plates), while freshly harvested primary human hepatocytes and HepG2 cells were, respectively, inoculated with 20,000 or 5,000 P. yoelii sporozoites per well (96-well plates) in a total volume of 50 μl. After inoculation, the culture plates were centrifuged for 10 min at 2,000 rpm in order to sediment the parasites and thereafter were incubated at 37°C under 5% CO_2_. Three hours later, the medium was replaced, and the cultures were returned to 37°C under 5% CO_2_.

The origin of the primary hepatocytes is indicated in the figure legend. For each biological replicate, distinct sporozoite batches and freshly thawed primary hepatocytes were used. The data from each point from these experiments was generally derived from three technical replicates (number of duplicate wells).

### Drug evaluations.

Different schemes were used to expose infected hepatocyte cultures to drugs.

• P. cynomolgi-infected simian primary hepatocytes were exposed from day 5 (D5) to D8 post-sporozoite inoculation to serial dilutions of primaquine (PQ) or chloroquine (CQ) alone or to PQ combined with variable doses of CQ (Fig. 1A to D).
• P. falciparum-infected human primary hepatocytes were exposed from D4 to D7 post-sporozoite inoculation with fixed doses of CQ (1.9 μM) and a serial dilution of PQ (Fig. 2A) or to fixed doses of CQ (1.9 μM) and serial dilutions atovaquone (ATO) (Fig. 2B).
• P. berghei-infected simian primary hepatocytes were exposed from 3 h to 48 h post-sporozoite inoculation to PQ with increasing doses of CQ (Fig. 2C) or to fixed doses of PQ (3, 10, and 30 μM) (Fig. 3A).
• P. yoelii infections in human primary hepatocytes or in HepG2 cell lines were treated from 3 to 48 h post-sporozoite inoculation with a fixed dose of PQ (0.91 μM) with increasing concentrations of CQ (Fig. 2D).
• Cultures not exposed to drugs served as negative controls.

Culture medium, with or without drugs, was changed daily for cultures infected by P. berghei or P. yoelii or every 48 h for those infected with P. cynomolgi or P. falciparum. The cultures were fixed at 48 h postinoculation for P. berghei and P. yoelii, at D7 for P. falciparum, and at D8 for P. cynomolgi.

The hepatic parasite exo-erythrocytic forms (EEFs) of P. cynomolgi, P. falciparum, and P. yoelii were then detected by microscopic examination of immunofluorescence using a mouse anti-P. falciparum HSP70 polyclonal antibody (1:2,000 in 1× phosphate-buffered saline [PBS]) that is also cross-reactive with P. cynomolgi and P. yoelii. Antibody binding was revealed with Alexa Fluor 488-conjugated goat anti-mouse immunoglobulin (Invitrogen). Parasites and cell nuclei were stained with 1 μg/ml 4,6-diamidino-2-phenylindole (DAPI, Sigma). Visible parasites were enumerated using a Leica DMI 4000 B fluorescence microscope with ×20 magnification. The person counting the parasites was blinded to the drug concentrations used.

For the P. berghei PbGFP ANKA, the EEF numbers were determined using a Cell Insight high-content screening platform equipped with Studio HCS software (Thermo Fisher Scientific) based on GFP fluorescence and DAPI staining, as described previously (19). The area of the EEFs observed was measured using the Cell Insight high-content screening platform (Thermo Fisher Scientific). Validation of the automatized counting was done by comparing sets of data from plates counted manually and with the HCS system. Calculation of the Pearson coefficient showed a robust correlation, close to 0.9 (0.893 ± 0.056).

Dose-response curves and 50% inhibition concentrations (IC_50_) were obtained by nonlinear regression with GraphPad Prism 5 software or ICEstimator software (www.antimalarial-icestimator.net) from normalized data compared to the untreated control. GraphPad Prism software and a *t* test were used for condition comparison purposes. A *P* value of 0.05 or less was considered to be statistically significant.

## Supplementary Material

Supplemental file 1
