# Supplementary material for: Chloroquine Potentiates Primaquine Activity against Active and Latent Hepatic Plasmodia Ex Vivo: Potentials and Pitfalls
Source: Antimicrob Agents Chemother. 2020 Dec 16;65(1):e01416-20. doi: 10.1128/AAC.01416-20 (PMC7927800; doi:10.1128/AAC.01416-20)
Supplement: Supplemental file 1 [file AAC.01416-20-s0001.pdf]

SUPPLEMENTARY FILE

Laurent Dembélé, Jean-François Franetich, Valérie Soulard, Nadia Amanzougaghene, Shahin Tajeri, Teun Bousema, Geert-Jan van Gemert, Roger Le Grand, Nathalie Dereuddre-Bosquet, J. Kevin Baird, Dominique Mazier, Georges Snounou

Chloroquine Potentiates Primaquine Activity Against Active and Latent Hepatic Plasmodia *Ex vivo*: Potentials and Pitfalls

Supplement to Fig 1C & 1D

Chloroquine potentiation of primaquine activity on *P. cynomolgi* hepatic stage parasites. Individual dose response curves for the four independent assays of PQ (x-axis) activity on the hepatic schizonts (Sch) or the putative hypnozoite (Hyp) of *P. cynomolgi* in primary simian hepatocytes (SH) in the presence or absence of a fixed CQ concentration. Each point was derived from data from 3 replica wells (error bars represent ± standard deviation). Figures 1C and 1D are based on this data.

Supplement to Figure 1C and 1D

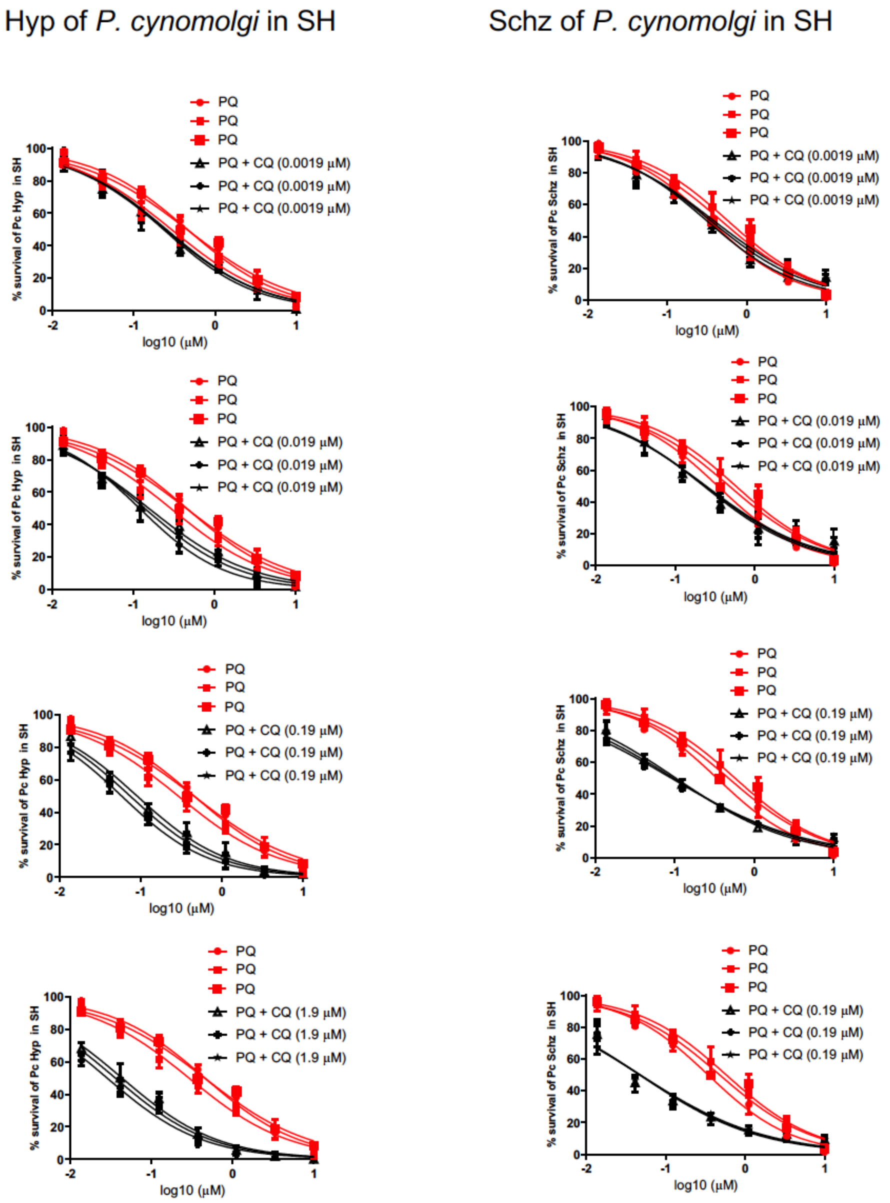

The IC50 values derived from the individual experiments in which the infected cultures were exposed to PQ in the absence or presence of 1.9 µM CQ are provided in the top Table. The average IC50 values derived from the four independent experiments for the schizonts, hypnozoites or all EEF are provided in the bottom Table.

Supplement to Figure 1D

Replactes	0µM CQ	1.9µM CQ
Exp1	0.3932	0.02028
Exp2	0.4728	0.01817
Exp3	0.4207	0.02906
Exp4	0.3819	0.02069

IC_{50} values obtained for four independent infected cultures treated with PQ in the absence or presence of 1.9 μM CQ.

PQ+CQ (µM)	Schz	Hyp	All EEFs
0	0.330495	0.526887	0.4171
0.001938	0.179525	0.359744	0.238
0.01938	0.129788	0.246954	0.1502
0.1938	0.014079	0.070703	0.0611
1.938	0.005712	0.021415	0.022

Average of the IC_{50} values obtained for the schizonts, hypnozoites, or all the EEF forms in the four independent cultures infected with P. cynomolgi and treated with PQ in the absence or presence of CQ.

Supplement to Fig 2C

Chloroquine potentiation of primaquine activity on the hepatic stages of *P. berghei*. Dose response curve of PQ (x-axis) activity on the hepatic forms (EEF) of *P. berghei* in primary simian hepatocytes (from batch SH30093L2) in the presence of increasing concentrations of CQ. An average of 130 EEFs to 150 EEFs were observed in each of the control wells (exposed from 3 h to 48 h post-sporozoite inoculation). The data is represented as curves with each point derived from four replica wells (error bars represent ± standard error). The raw data for this experiment is also presented.

Supplement to Figure 2C

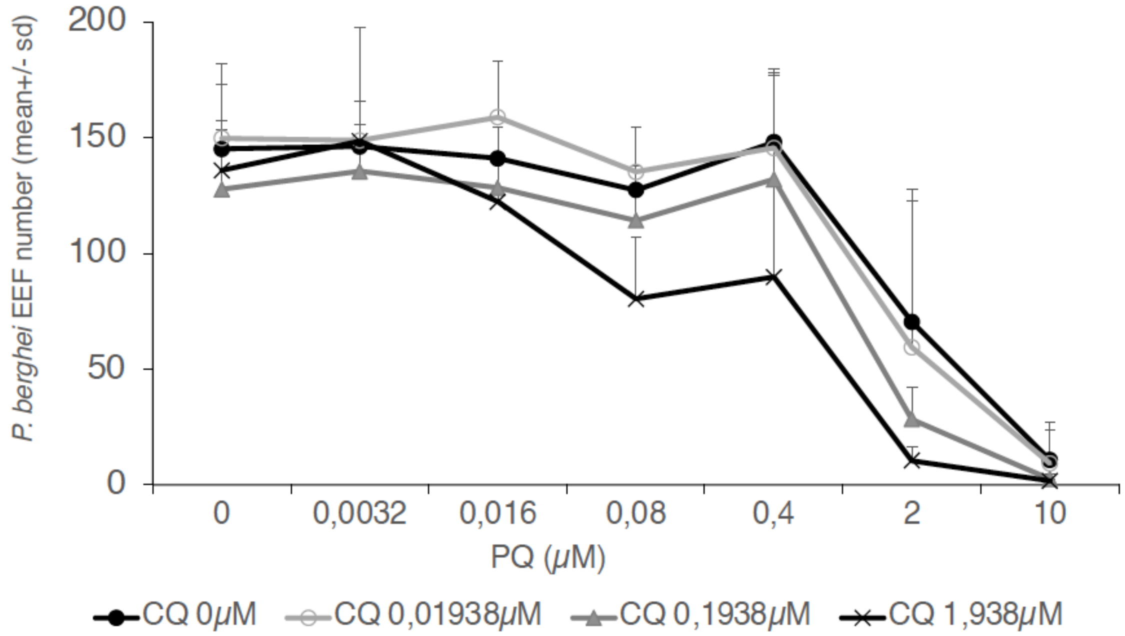

Supplement to Fig 2D

Chloroquine potentiation of primaquine activity on the hepatic stages of *P. yoelii*. Individual dose response curve of PQ (x-axis) activity on the hepatic forms (EEF) of *P. yoelii* in presence of increasing concentrations of CQ, either in primary human hepatocytes (HH) or in the HepG2 hepatocarcinoma line (exposed from 3 h to 48 h post-sporozoite inoculation). An average of 187 EEFs in HH, and 228 EEFs in HepG2 were observed in each of the control wells. The data presented for *P. yoelii* are derived from three independent biological replicates in which each point derived from three replica wells (error bars represent ± standard deviation) two technical replicates (error bars represent ± standard deviation). The human primary hepatocytes used for the *P. falciparum* and *P. yoelii* experiments were derived from three distinct donors.

Supplement to Figure 2D

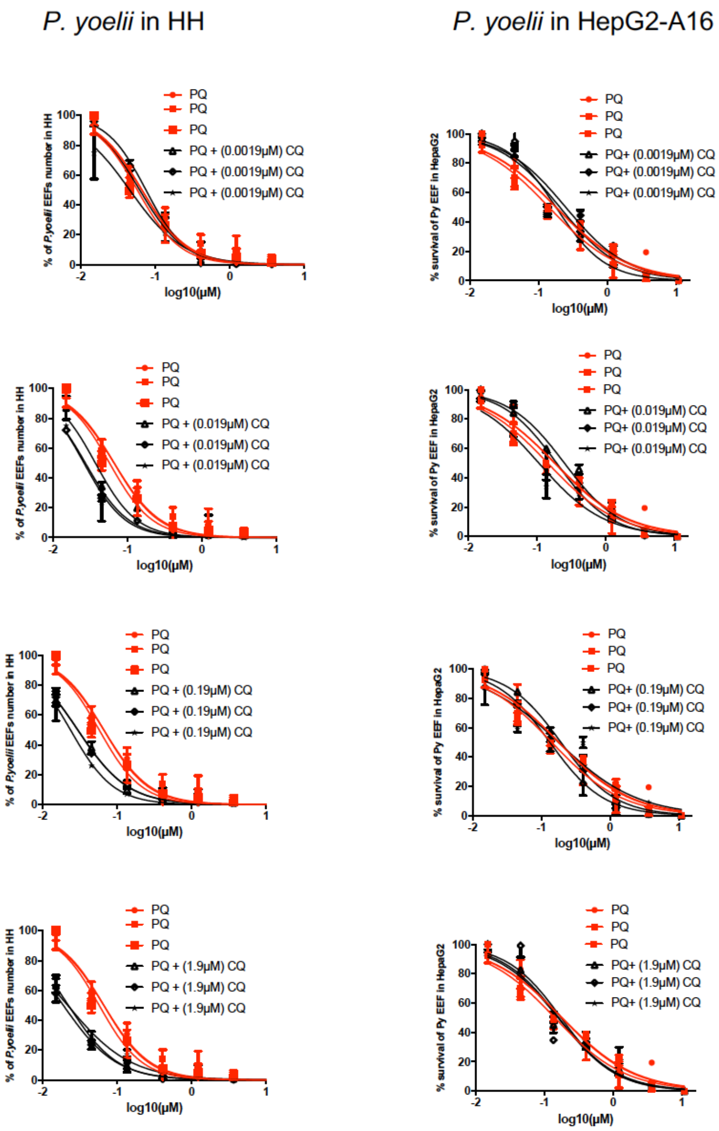

Supplement to Fig 3A

3A-1 and 3A-2: Hepatocyte nuclei observed in *P. berghei* PbGFP-infected cultures treated with two batches of primaquine (PQ #MKBP5004V and PQ #01126ED) at 3, 10 or 30 µM, from 3 hours post infection to fixation at D2. Each bar represented the mean number of hepatocyte nuclei in the three replica wells for each of the three distinct batches of simian primary hepatocytes isolated from three different *M. fascicularis* monkeys: SH30161L1, grey bars; SH30093L2, black bars; and SH30790L1, blank bars. Of note, the presence of 10 µM methylene blue in the SH30161L1 culture has no influence on the viability of the hepatocytes (compare 3A-1 and 3A2).

Supplement to Figure 3A-1

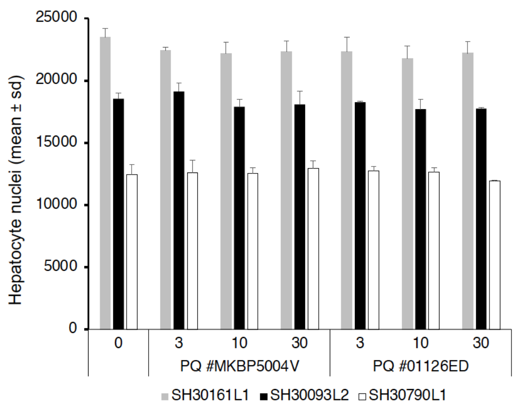

PQ effect on 3 distinct simian primary hepatocyte batches
Nuclei counts/well in the absence of MB

Supplement to Figure 3A-2

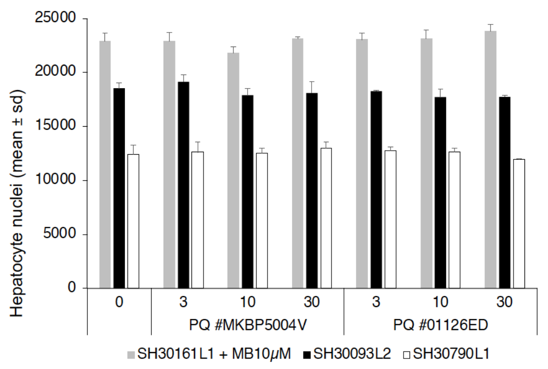

Nuclei counts/well for batch SH30161L1 in the presence of 10 µM MB
NB: Data for batches SH30093L2 and SH30790L1 are from wells unexposed to MB

3A-3: Number of *P. berghei* PbGFP hepatic forms (EEF) in three distinct batches of simian primary hepatocytes isolated from three different *M. fascicularis* monkeys (SH30161L1, grey bars; SH30093L2, black bars; and SH30790L1, blank bars) exposed from 3 hours post infection to fixation at D2 to two batches of primaquine (PQ #MKBP5004V and PQ #01126ED) at 3, 10 or 30 µM. Of note, the abnormally low count in the control well of SH30161L1 (due to accidental partial dehydration of the plate), which precluded the use of the data derived from this experiment.

Supplement to Figure 3A-3

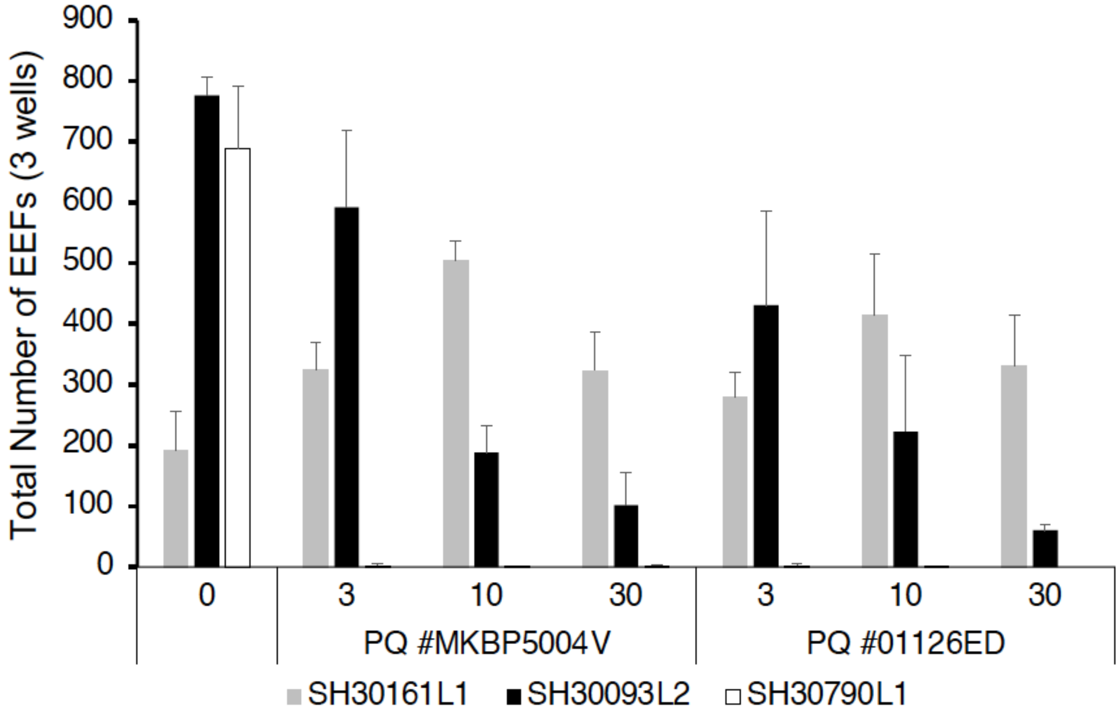

Number of parasite forms observed in the absence of MB for all batches. Partial accidental dehydration of the plate affected the SH30161L1 wells, and in particular those of the no drug controls.

Supplement to Fig 3B

The experimental scheme used for the four independent experiments in each of which one the four distinct batches of PQ was tested.

Supplement to Figure 3B

Experiment 1

Hep + spz batch 1 + #P2940000

Experiment 2

Hep + spz batch 2 + #160393

Experiment 3

Hep + spz batch 3 + #1561507

Experiment 4

Hep + spz batch 4 + #P9504

A distinct and independent experiment was conducted for each PQ lot. In each, freshly thawed out simian primary hepatocytes (derived from the same animal) were infected with a different batch of *P. cynomolgi* sporozoites, and treated with one of the four different batches of primaquine.
